# Towards phenotyping stroke: Leveraging data from a large-scale epidemiological study to detect stroke diagnosis

**DOI:** 10.1371/journal.pone.0192586

**Published:** 2018-02-14

**Authors:** Yizhao Ni, Kathleen Alwell, Charles J. Moomaw, Daniel Woo, Opeolu Adeoye, Matthew L. Flaherty, Simona Ferioli, Jason Mackey, Felipe De Los Rios La Rosa, Sharyl Martini, Pooja Khatri, Dawn Kleindorfer, Brett M. Kissela

**Affiliations:** 1 Department of Biomedical Informatics, Cincinnati Children’s Hospital Medical Center, Cincinnati, Ohio, United States of America; 2 Department of Pediatrics, College of Medicine, University of Cincinnati, Cincinnati, Ohio, United States of America; 3 Department of Neurology and Rehabilitation Medicine, University of Cincinnati, Cincinnati, Ohio, United States of America; 4 Department of Emergency Medicine and Neurosurgery, University of Cincinnati, Cincinnati, Ohio, United States of America; 5 Department of Neurology, Indiana University, Indianapolis, Indiana, United States of America; 6 Baptist Health Neuroscience Center, Miami, Florida, United States of America; 7 Michael E. DeBakey VA Medical Center, Houston, Texas, United States of America; University of Illinois-Chicago, UNITED STATES

## Abstract

**Objective:**

1) To develop a machine learning approach for detecting stroke cases and subtypes from hospitalization data, 2) to assess algorithm performance and predictors on real-world data collected by a large-scale epidemiology study in the US; and 3) to identify directions for future development of high-precision stroke phenotypic signatures.

**Materials and methods:**

We utilized 8,131 hospitalization events (ICD-9 codes 430–438) collected from the Greater Cincinnati/Northern Kentucky Stroke Study in 2005 and 2010. Detailed information from patients’ medical records was abstracted for each event by trained research nurses. By analyzing the broad list of demographic and clinical variables, the machine learning algorithms predicted whether an event was a stroke case and, if so, the stroke subtype. The performance was validated on gold-standard labels adjudicated by stroke physicians, and results were compared with stroke classifications based on ICD-9 discharge codes, as well as labels determined by study nurses.

**Results:**

The best performing machine learning algorithm achieved a performance of 88.57%/93.81%/92.80%/93.30%/89.84%/98.01% (accuracy/precision/recall/F-measure/area under ROC curve/area under precision-recall curve) on stroke case detection. For detecting stroke subtypes, the algorithm yielded an overall accuracy of 87.39% and greater than 85% precision on individual subtypes. The machine learning algorithms significantly outperformed the ICD-9 method on all measures (P value<0.001). Their performance was comparable to that of study nurses, with better tradeoff between precision and recall. The feature selection uncovered a subset of predictive variables that could facilitate future development of effective stroke phenotyping algorithms.

**Discussion and conclusions:**

By analyzing a broad array of patient data, the machine learning technologies held promise for improving detection of stroke diagnosis, thus unlocking high statistical power for subsequent genetic and genomic studies.

## Introduction

Stroke is the fifth leading cause of death in the US and is a major cause of adult disability.[1] Patients with stroke require expensive long-term rehabilitation care, resulting in an annual cost of over $33 billion nationally.[1] The main pathological subtypes of stroke include ischemic stroke, hemorrhagic stroke, and transient ischemic attack (TIA). Understanding clinical causation of stroke and its subtypes is critical for the planning, implementation, and evaluation of patient treatments. In particular, it will enable development of stroke phenotypes, which is the first step toward more powerful genetic and genomic studies that can lead to a better understanding of stroke etiology.[2–4] However, determination of stroke and its subtypes requires integration of multiple demographic, clinical, diagnostic, and imaging features; consequently, there is great variability between individual patients.[5–12]

Previous efforts have been made to identify predictors associated with stroke diagnosis. Medical history of hypertension, hyperlipidemia, obesity, diabetes mellitus, and atrial fibrillation have been commonly recognized as risk factors associated with stroke.[6, 9, 13–16] Computed tomography (CT) and magnetic resonance imaging (MRI) are routinely used in the diagnostic work-up of stroke patients. As new technologies of image processing have been introduced over time, imaging patterns have been increasingly adopted as “image markers” to facilitate stroke diagnosis.[17–19] In addition to clinical characteristics, patient demographics, family history, and substance use behaviors are considered influential factors on their risk of stroke.[6, 11, 20] Despite these findings, no single factor or group of factors would make a definite diagnosis. Rule-based approaches have been developed to heuristically combine the predictors to identify stroke cases, but large variability in reported sensitivities and specificities exists for the assessments.[21, 22] To detect stroke subtypes, current studies typically rely on International Classification of Diseases (ICD) codes or death certificate data. However, they are limited by precisions ranging from 6% to 97% across study designs and stroke subtypes.[23–28] Physician review of patients’ complicated medical records remains the gold-standard method of ascertaining stroke diagnosis, and the process is labor intensive and expensive.[29, 30]

Machine learning (ML) is a methodology of data analytics that utilizes computerized algorithms to identify the relation between, and make prediction on, sets of data. By iteratively learning from example inputs (i.e., training data), ML algorithms identify hidden insights of the data and generate predictions on unseen examples (i.e., test data). In the literature of stroke research, ML technologies have been applied to identify stroke cases,[15, 31] predict stroke outcomes (e.g., mortality and recurrent stroke),[32–34] and evaluate therapy outcomes.[35, 36] Nevertheless, most of the studies have been limited to small patient cohorts (fewer than 200 samples), explored limited predictors, and did not have statistical power to discover relationships among a larger set of risk factors. A handful of studies utilized larger datasets (about 3000) to develop stroke detection models.[37, 38] However, their optimal accuracy plateaued at less than 75%.[37] In particular, none of the studies investigated the detection of stroke subtypes. Because ascertainment of stroke subtypes requires integration of findings from multiple clinical assessments and diagnostic tests,[39–41] the complexity and accuracy in detecting individual subtypes can vary dramatically.[25, 26] Additional study is therefore required to evaluate the effectiveness of ML technologies on stroke subtype detection.

Epidemiology studies collect a tremendous amount of multi-site samples with corresponding demographic and clinical data.[5, 42–44] In particular, some studies utilize physician review of the electronic health record (EHR) data to confirm stroke diagnosis for improved ascertainment accuracy.[43] By utilizing a comprehensive list of clinical data collected from such population-based metropolitan study, we investigated ML methodology to detect stroke diagnosis.

## Objective

Our long-term objective is to develop a phenotyping algorithm that retrospectively identifies stroke cases across institutions to support genetic and genomic research. Because genetic and genomic studies typically require a case cohort of high purity (represented with a precision of 95%), we aim to establish a ML approach to detect stroke diagnosis with high precision and adequate recall. The specific aims of this study are: 1) to develop a ML approach to detect stroke cases and subtypes based on a broad array of hospitalization data; 2) to assess algorithm performance and predictors on real-world data collected from a large-scale epidemiology study of stroke in the US; and 3) to identify directions for future development of stroke phenotypic signatures. The study is the first, known to us, to investigate detection of multiple stroke subtypes in a large-scale via ML technologies.

## Materials and methods

We utilized all hospitalization events collected from the Greater Cincinnati/Northern Kentucky Stroke Study (GCNKSS), a large-scale, population-based epidemiology study that measures temporal trends in stroke incidence rates in a population of 1.3 million.[43] The study was approved by the institutional review boards of participating hospitals (University of Cincinnati, Tri-Health, the Jewish Hospital and Mercy Hospital System, the Christ Hospital, and the St. Elizabeth Healthcare) and a waiver of individual consent was authorized (Study ID: 2013–3959 04061501).

**Fig 1:**
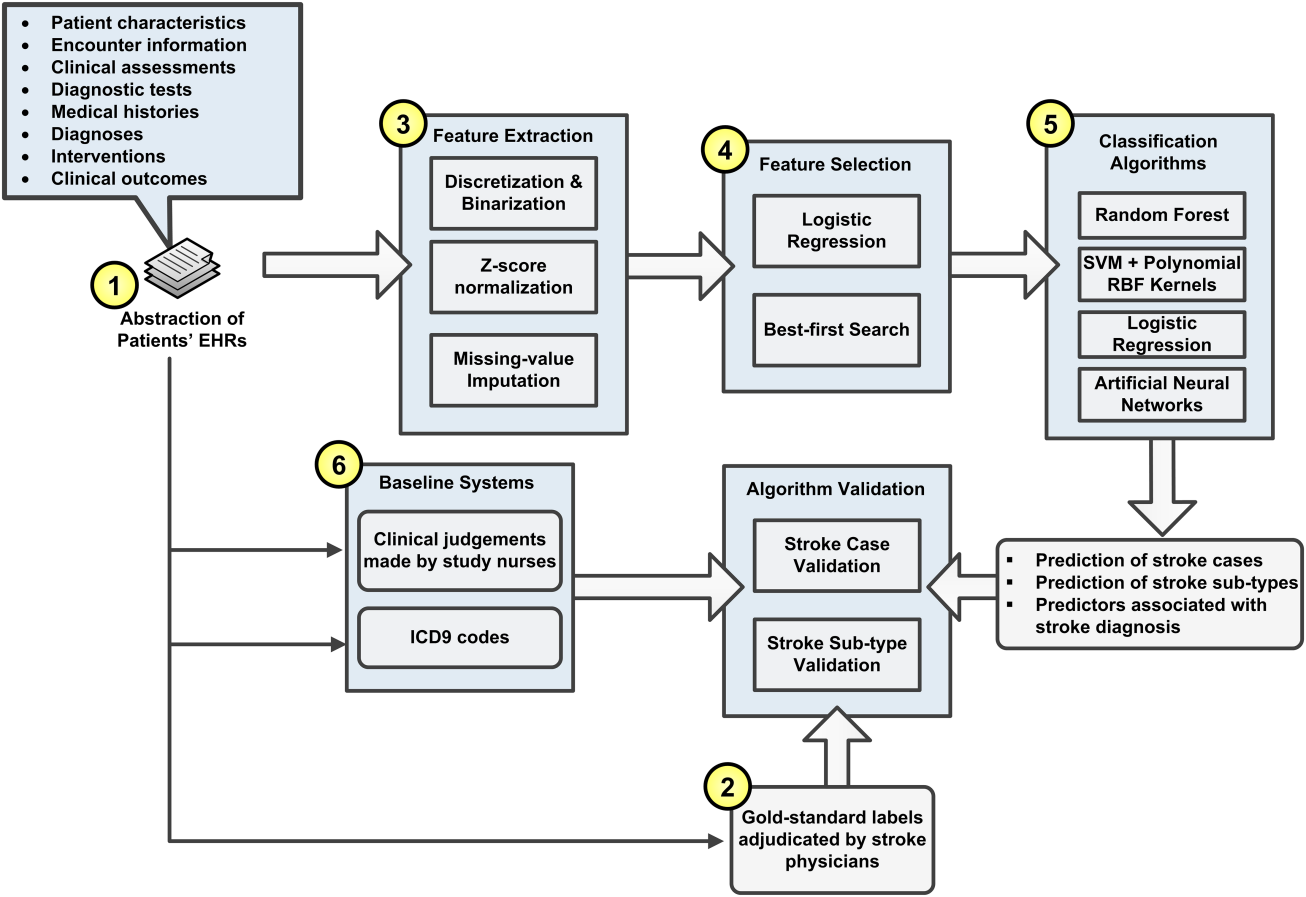
The overall processes of the study.

Fig 1 diagrams the overall processes of the study. We first extracted clinical variables and stroke adjudications for all hospitalization events from the GCNKSS data (processes 1 and 2 in Fig 1). ML technologies were then applied to build stroke detection models with three steps: 1) features were generated from the variables and were normalized (process 3), 2) feature selection was applied to select predictive features for model construction (process 4), and 3) a variety of ML algorithms were developed to detect stroke diagnosis based on the selected features (process 5). Finally, the performance of ML models was assessed and compared with that of ICD-9 method and human experts (process 6).

### Stroke events and patient EHR data

The GCNKSS collected and ascertained all potential stroke events that occurred among residents of the study region in 2005 and 2010. The GCNKSS first identified hospitalization events with potential stroke-related diagnoses from all 19 regional hospitals using ICD-9-CM codes (primary or secondary discharge diagnoses) of 430–438 that match the World Health Organization definition of stroke.[45] Detailed information from patients’ EHRs was then abstracted for each event by trained research nurses (process 1 in Fig 1). We selected all 316 structured variables that specified patients’ characteristics, encounter information, physiological status, diagnostic tests, medical histories, hospital diagnoses, interventions and clinical outcomes. The variables were categorized into 19 sets, which are summarized in Table 1. The description of each variable is presented in S1 Table. Because our goal was to retrospectively identify stroke cases, we leveraged all available information from a patient’s hospitalization, including ICD discharge codes, interventions, and clinical outcomes.

**Table 1:** Summary of the variables used in the study.

Variable Category	Number of Variables	Description
DEMO	6	Patient demographics, including age, sex, race, ethnicity, marital status and employment status
SU	11	Patients’ history of substance use (smoking, alcohol and street drugs)
VI	4	Visit information at time of admission (e.g., type of first medical contact, type of visited institution)
ED	13	Evaluations (e.g., blood pressure, Glasgow Coma Scale) performed in the emergency department
SE	29	Stroke-related evaluations (e.g., NIH stroke scale)
SS	20	Signs and symptoms that caused a patient to seek medical attention (e.g., weakness, headache, speech and vision)
CT/MRI	24	CT or MRI performed (Yes/No) and, if so, the findings (e.g., normal, acute infarct, intracerebral hemorrhage)
ANG	6	MRA, CTA, or cerebral angiography performed (Yes/No) and, if so, the findings (e.g., normal/abnormal)
CU	2	Carotid ultrasound performed (Yes/No) and, if so, the findings (e.g., normal/abnormal)
ECHO	19	Echocardiogram performed (Yes/No) and, if so, the findings (e.g., cardiomyopathy Yes/No)
EKG	16	Electrocardiogram performed (Yes/No) and, if so, the findings (e.g., normal/abnormal)
LAB	14	Laboratory results collected during hospitalization (e.g., white blood cell count, glucose level, total cholesterol)
MH	52	General medical history prior to hospitalization (e.g., history of hypertension Yes/No)
SH	18	History of stroke prior to hospitalization (e.g., ischemic stroke Yes/No)
ICD9	1	Primary and secondary ICD-9 codes on patients’ discharge lists
DX	47	Complications and new diagnoses during hospitalization (e.g., pain, seizure, cardiac arrest Yes/No)
IT	13	Interventions performed (e.g., aneurysm clipping/coiling, clot evacuation Yes/No)
TH	15	Therapies performed (e.g., physical, occupational or speech therapy Yes/No)
OC	6	Clinical outcome of hospitalization (e.g., disposition at discharge, modified Rankin Scale)

### Gold-standard stroke diagnosis

Ten stroke physicians were available to adjudicate study abstracts. Each abstract was reviewed by at least one stroke physician to determine whether the event was a stroke case and, if so, the stroke subtype (process 2 in Fig 1). Complicated events (35.1% of the collected events) were adjudicated by at least two physicians through group discussion to ensure the accuracy of diagnosis. The adjudicators had rigid criteria to determine stroke cases and subtypes,[43] but they were allowed to use their clinical judgment to clarify events (e.g., MRI negative for stroke but clinical symptoms and history consistent with stroke diagnosis could be called a case). For our study, we maintained the case criteria without exception: an event was labeled as a stroke case only if it met the case criteria. The event labels adjudicated by physicians were used to train and evaluate the ML algorithms.

### Detecting stroke diagnosis with ML technologies

#### Feature extraction

We followed the methodology used in our earlier studies to process the clinical variables.[46–48] All nominal variables (e.g., sex, ICD-9 codes) were converted to binary features using dummy variable coding.[49] We then used two methods to discretize and normalize numerical variables. The National Institute of Health Stroke Scale (NIHSS) and Glasgow Coma Scale were discretized into categories based on clinical classification.[50, 51] For example, the NIHSS was discretized into no symptoms (0), mild (1–4), moderate (5–14), severe (15–24), and very severe (25–42) to stratify stroke severity.[50] The real-valued vital signs were discretized into “normal” and “out of normal range”.[52] The remaining variables, including age and laboratory results, were normalized using z-score normalization.[53] Because a patient might not take all diagnostic tests and assessments during hospitalization, the event samples could have missing values on certain variables. To alleviate the influence of missing data, we implemented unique-value imputation and grand mean and mode imputation based on their computational efficiency and performance in ML tasks.[54, 55] For each nominal variable, we created a unique category representing “unknown” for missing values. For a numerical variable, we replaced the missing values with the variable’s mean (for continuous variables) or mode (for discrete variables) derived from the data.

#### Feature selection

The feature extraction yielded a large number of features for model construction. To reduce noise and avoid overfitting, we implemented a wrapper-based feature selection using logistic regression (LR) and best first search.[56, 57] The feature selection also provided a better insight of individual variables contributing to stroke diagnosis.

In each iteration, features from a category variable (Table 1) was added to the LR for training and testing to determine the top-performing category. The process was repeated until all 19 categories were added. The optimal feature set was chosen as the point at which additional features did not increase predictive performance. Note that some ML algorithms (e.g., random forest) inherently eliminate irrelevant features during model training, and they might not benefit from feature selection. As such, whether using the original or the optimal feature sets was tuned for individual ML algorithms based on the cross-validation performance.

#### Stroke case detection

We formatted detection of stroke cases as a binary-class classification and implemented four ML classifiers: 1) LR, a direct probability model that measures the linear relationship between features and stroke diagnosis;[58] 2) support vector machines with polynomial (SVM-P) and radial basis function (SVM-R) kernels, which construct hyperplanes in linear and non-linear feature spaces to classify stroke cases and non-stroke “controls”;[59] 3) random forest (RF), which uses a multitude of decision trees to learn a highly irregular combination of features;[60] and 4) artificial neural networks (ANNs) that comprise three layers of LR models to learn non-linear patterns among features.[58] We chose these classifiers to allow for the possibility of linear and non-linear relationships between features and stroke diagnosis.

The classifiers output predictive values (-∞, +∞) to represent the possibility of stroke diagnosis. If a predictive value was positive, we assigned +1 to the output suggesting a stroke case. Otherwise, we assigned -1 suggesting a non-stroke “control”. Given that the values output by ANNs ranged between 0 and 1, we set the threshold to 0.5 for ANNs.

#### Stroke subtype detection

We modeled stroke subtype detection as a task of four-class (ischemic stroke, hemorrhagic stroke, TIA, and non-stroke “control”) classification. The RF and ANNs are natural multiclass classifiers, and they can predict the possibilities of classes simultaneously. The LR and SVM were extended to multiclass setting using the one-versus-all approach,[58] which trained a single classifier per class, with the samples of that class as cases and all other samples as controls. After training, it applied all classifiers to a test example and predicted the class for which the corresponding classifier output the highest predictive value.

#### Coping with imbalanced data

The distribution of stroke events in the real-world data was unbalanced, which could cause prediction bias and compromise the performance of ML algorithms.[61] Because the majority of abstracted events were stroke cases, the ML algorithms might predict all events as cases; this would achieve high accuracy, but would sacrifice other measures such as precision. To address this issue, we adopted adaptive synthetic sampling (ADA-SYN) to oversample minority class (e.g., non-stroke “control”) in the training data.[62] The algorithm adaptively synthesized different numbers of samples from each minority example until the classes reached similar sizes. The balanced data were then used to train the ML algorithms. Similar to feature selection, the ADA-SYN sampling was integrated into the cross-validation process.

### Baseline systems

**Fig 2:**
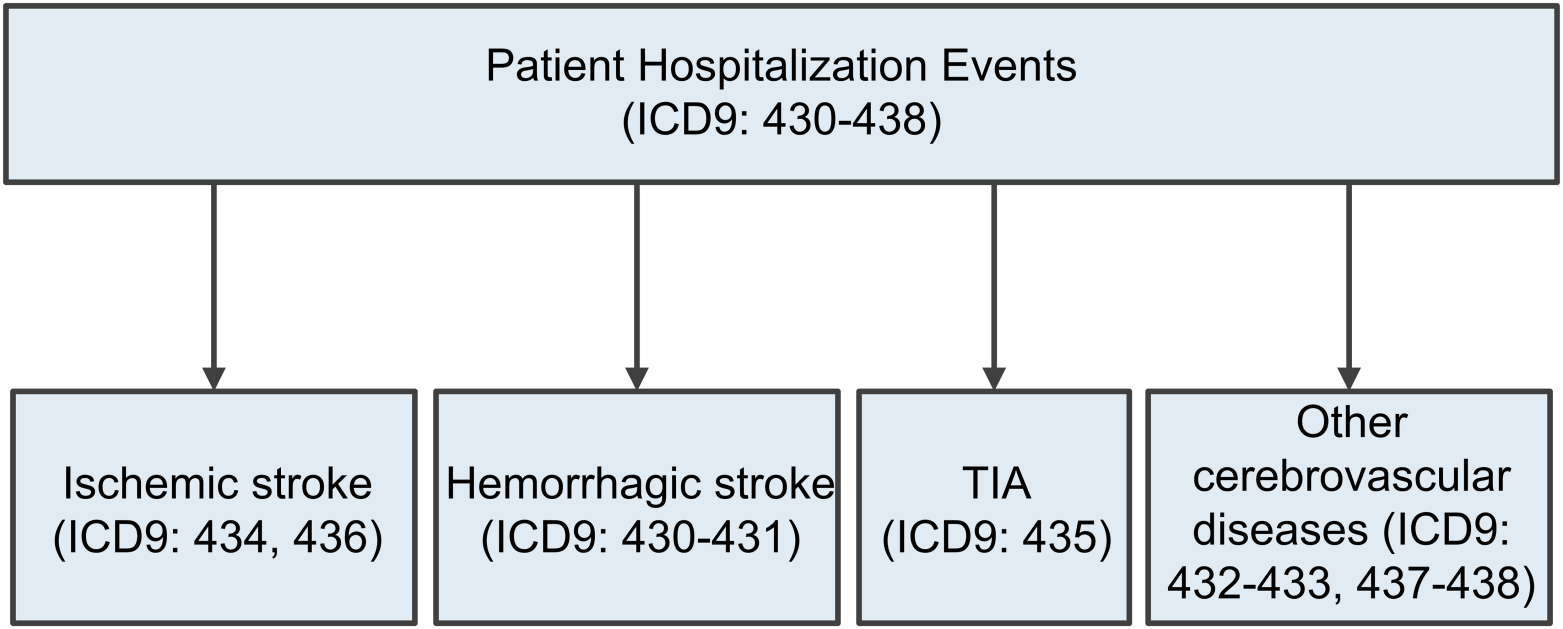
The ICD-9 coded baseline.

We implemented two baseline systems to compare with the ML algorithms (process 6 in Fig 1). The first baseline was an ICD9-coded method (denoted by ICD9) because phenotype algorithms frequently use ICD codes to identify qualified cases.[26, 63] The method was developed to identify high-precision stroke cohorts, and its logic is illustrated in Fig 2.[26] In the GCNKSS the abstractors (trained research nurses) also provided their judgments of stroke diagnosis for each event. We used these clinical judgments as the second baseline (denoted by CLIN) that simulated the decision-making of research nurses on stroke diagnosis.

### Experiments

#### Evaluation metrics

We adopted five customary evaluation metrics to assess algorithm performance: 1) Accuracy = (True positives+True negatives)/Total events (denoted by ACC); 2) Precision = True positives/(True positives+False positives) (denoted by P); 3) Recall = True positives/(True positives+False negatives) (denoted by R); 4) F-measure = 2PxR/(P+R) (denoted by F); and 5) the area under receiver operating characteristics curves, which measures balance between recall and specificity (denoted by AUC).[64–66] Because the goal of this study was to identify high-precision stroke cohorts with adequate recall, we also generated precision-recall curves and measured the area under the curve (denoted by AUC-PR) to assess balance between precision and recall.[67]

#### Experiment setup

We performed a stratified random sampling based on number of events for each stroke subtype to split the data into two sets, 80% for training and development and 20% for testing and error analysis. Two iterations of ten-fold cross-validation were applied on the training set to select features and tune model parameters. Both cross-validation processes used the same data partition. The first cross-validation was applied to perform feature selection and generate the optimal feature set. The second cross-validation was used to tune hyper-parameters of the ML classifiers, including cost parameters (C) for LR, SVM-P, SVM-R and ANN (screened at 2 increments from 2^−10^ to 2^16^), optimal degree for SVM-P (screened from 1 to 6), parameter *γ* for SVM-R (screened from 2^−15^ to 2^5^), number of trees for RF (screened from 2^2^ to 2^11^), and number of neuros for ANNs (screened at 20 increments from 10 to 100). Whether using ADA-SYN sampling and the optimal feature set was also tuned during the second cross-validation process. Finally, the ML classifiers with optimal parameters were applied to the test data for performance comparison and error analysis.

For stroke case detection, events with a definite stroke diagnosis were labeled +1, and events without a stroke diagnosis were labeled -1. The event labels were then used to train and evaluate the ML algorithms. Feature selection was performed to identify predictive variables. All evaluation metrics were used, and we adopted the AUC-PR as the primary measure.

**Fig 3:**
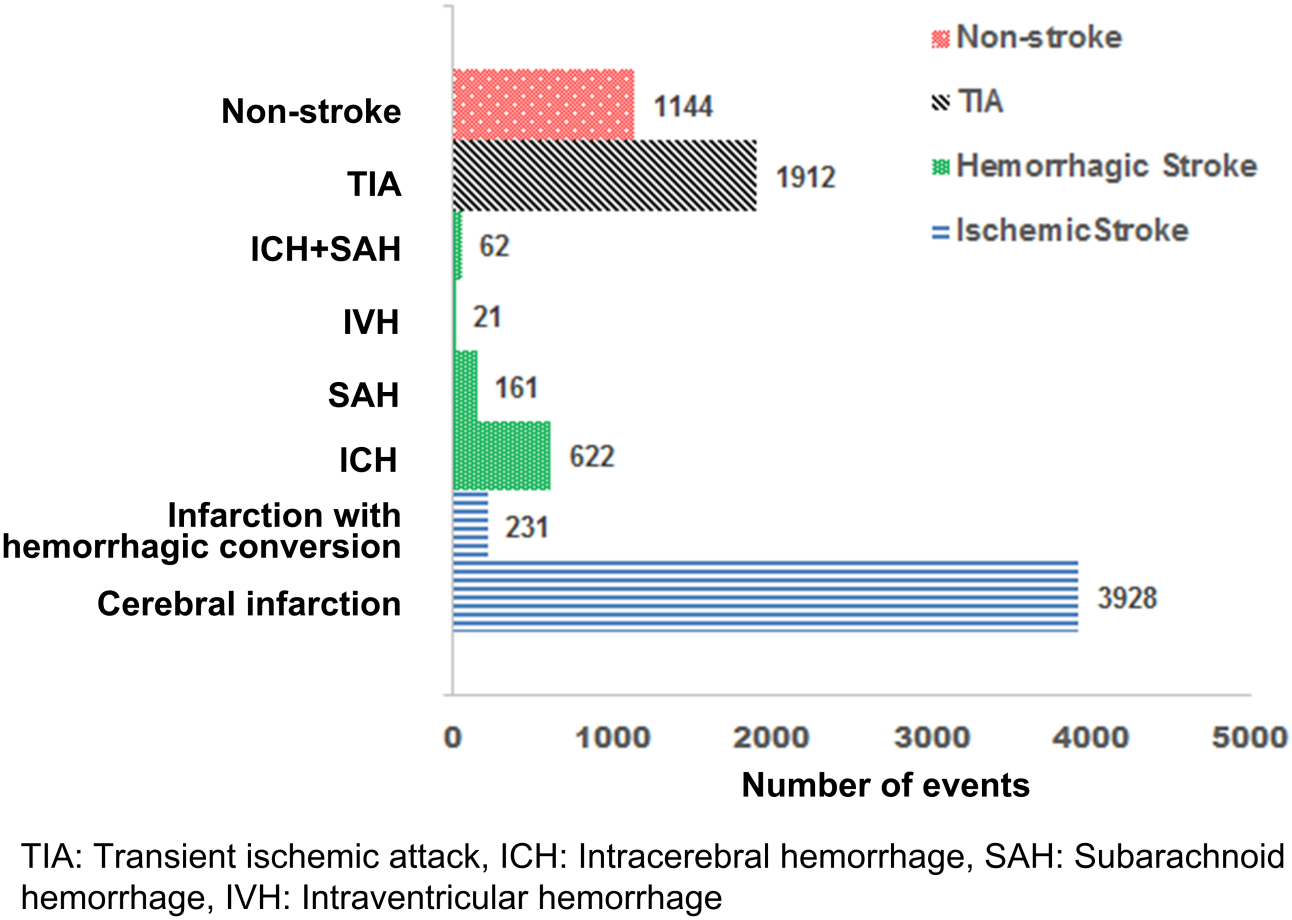
The event distribution of stroke subtypes among the four categories.

For stroke subtype detection, events were grouped into four categories (ischemic stroke, hemorrhagic stroke, TIA, and non-stroke “control”, Fig 3) based on their subtypes. They were then labeled 1–4 to train and evaluate the algorithms. The optimal feature set was inherited from stroke case detection that captured informative variables for all subtypes. We did not perform feature selection in the multiclass setting because the small sample sizes of minority classes (e.g., hemorrhagic stroke) could cause overfitting during feature selection and propagate errors to the classifiers.[68, 69] For evaluation we reported overall accuracy, and precision, recall, and F-measure on each category. We also compared confusion matrices between different algorithms. The accuracy was adopted as the primary measure. We did not assess AUC and AUC-PR because they were primarily designed for binary classification.

#### Statistical analysis

Our primary outcome was to demonstrate that using the ML approach would detect stroke diagnosis more accurately, compared with baseline methods (ICD9 and CLIN). To this end, the statistical significance of the difference between systems’ performances was assessed and reported using paired T-test.[70]

In our experiments the ML algorithms, evaluation metrics, and statistical analyses were implemented using MATLAB Version 2014a.[71]

## Results

### Descriptive statistics of the data set

The study personnel reviewed a total of 8,131 events, of which 6,987 samples (85.9%) were adjudicated to be stroke cases. We excluded 50 samples (0.6%) due to undetermined stroke subtypes. Fig 3 depicts the event distribution of stroke subtypes among the four categories. After stratified sampling and feature extraction, the training set contained 6,463 samples (3,327/692/1,529/915 ischemic/hemorrhagic/TIA/non-stroke) with 1,071 unique features. The test set had 1,618 samples (832/174/383/229 ischemic/hemorrhagic/TIA/non-stroke) with 994 features. In total there were 1,091 features in the data set.

### Results of feature selection

**Fig 4:**
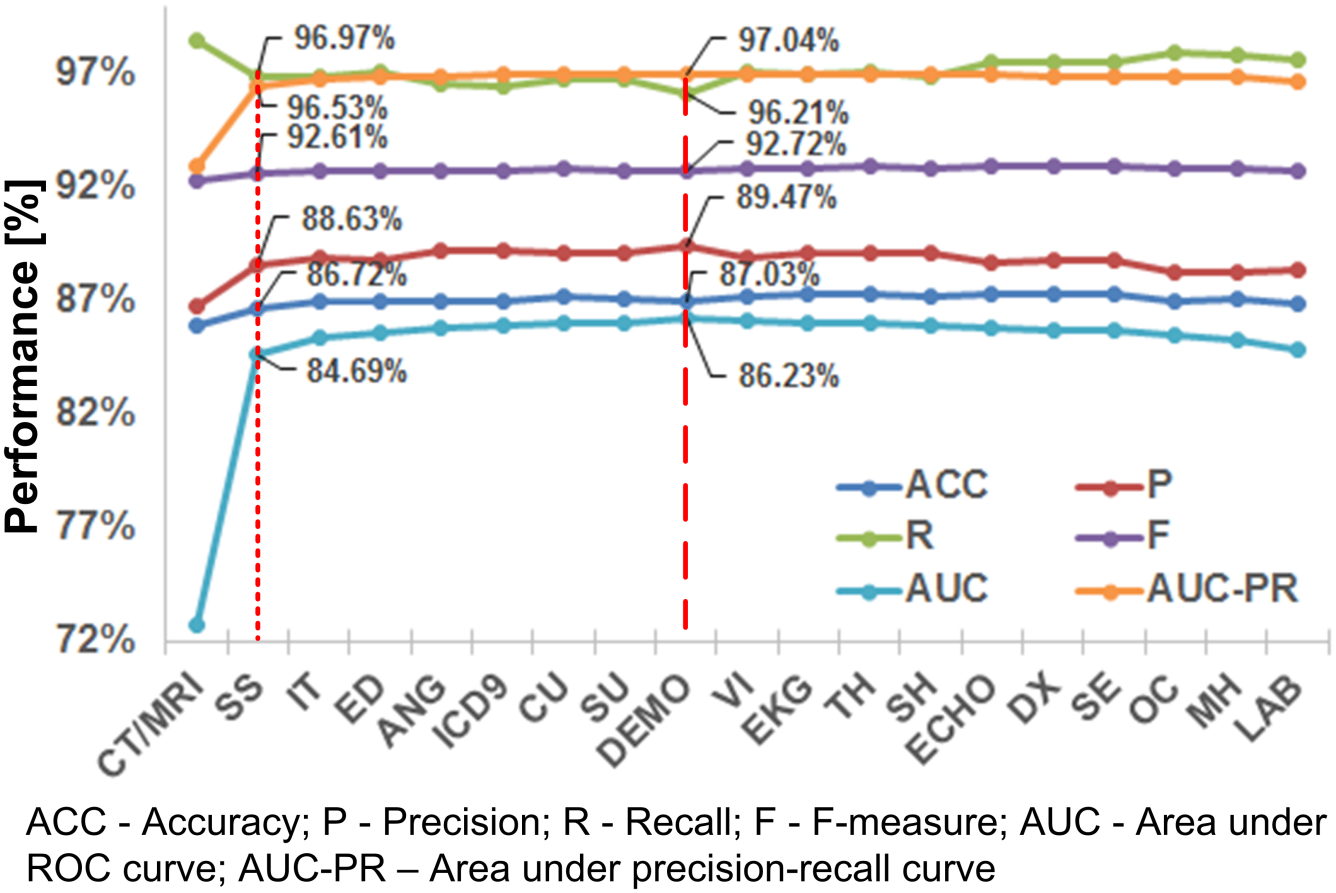
The performance curves when adding the variable sets (Table 1).

Fig 4 shows the performance curves on cross-validation for each incremental variable set addition. All performance measures except recall increased and then stabilized. The best AUC-PR and AUC achieved by feature selection were 97.04% and 86.23% respectively (dash line in Fig 4). The optimal feature set included CT/MRI findings (CT/MRI), signs and symptoms (SS), interventions (IT), ED assessments (ED), findings from angiography (ANG) and carotid ultrasound (CU) tests, ICD-9 codes (ICD9), substance use characteristics (SU), and demographics (DEMO). Using only CT/MRI and SS achieved an AUC-PR/AUC of 96.53%/84.69% (dotted line), which was close to the optimal performance.

### Performance of stroke case detection

**Fig 5:**
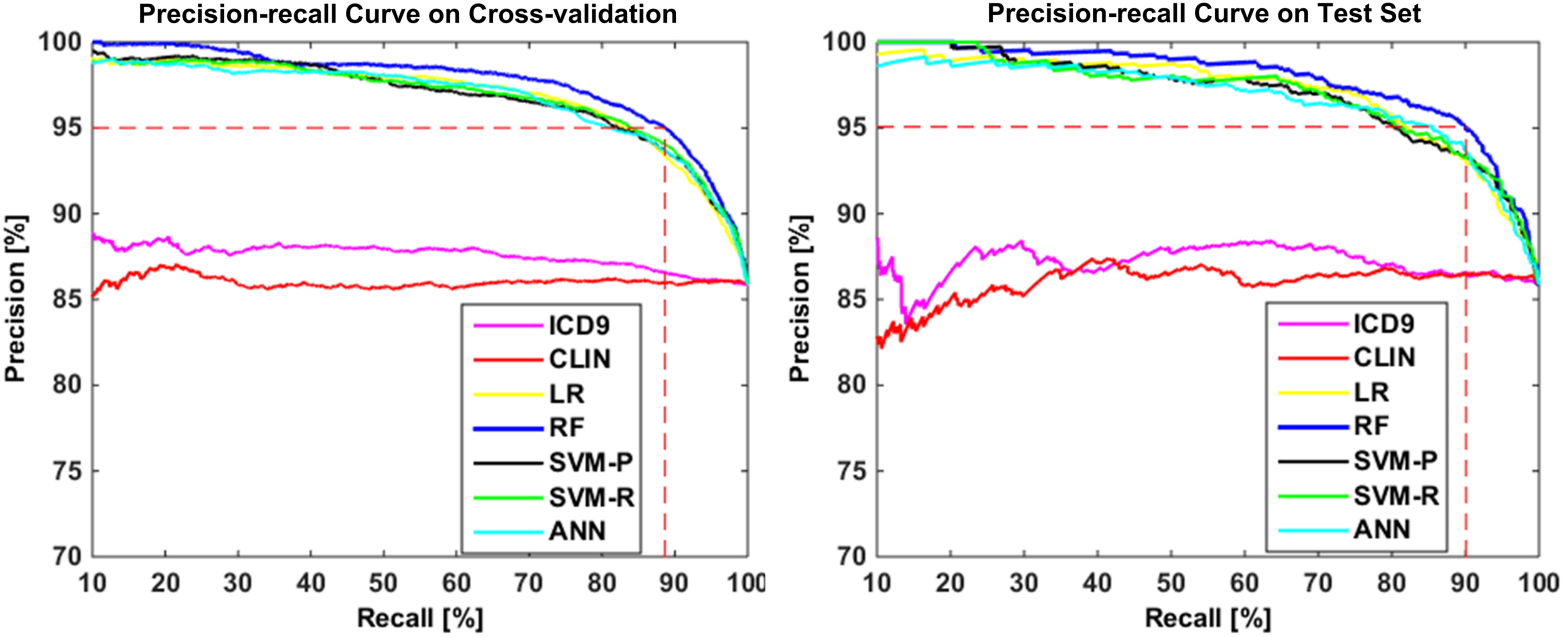
Precision-recall curves generated by the algorithms.

Table 2 shows the performance of different classification algorithms on detecting stroke cases. Compared with the ICD9 baseline, the ML classifiers performed significantly better on all measures (Table 3). They also outperformed research nurses (CLIN) on all measures except recall. Fig 5 plots precision-recall curves generated by the algorithms. The best curves were generated by the RF, with AUC-PR of 97.54% on cross-validation and 98.01% on the test set.

**Table 2:** Performance of different classification algorithms for stroke case identification.

**Measure**	**Cross Validation Performance [%]**						
	**ICD9**	**CLIN**	**LR**	**SVM-P**	**SVM-R**	**RF**	**ANN**
ACC	60.45	85.41	87.17	87.56	**88.07**	87.56	87.35
P	87.96	86.06	88.96	90.26	90.32	**92.75**	91.25
R	62.47	**99.05**	97.11	95.87	96.45	92.81	94.31
F	73.05	92.10	92.86	**92.97**	93.28	92.78	92.75
AUC	55.83	50.60	86.11	85.93	86.41	**88.02**	85.98
AUC-PR	87.91	83.29	97.15	96.81	96.86	**97.54**	96.74
**Measure**	**Test Set Performance [%]**						
	**ICD9**	**CLIN**	**LR**	**SVM-P**	**SVM-R**	**RF**	**ANN**
ACC	61.68	85.85	87.21	87.89	**88.38**	88.57	86.90
P	88.41	86.39	89.40	90.94	90.99	**93.81**	91.59
R	63.72	**99.14**	96.54	95.39	95.97	92.80	93.31
F	74.06	92.32	92.84	93.11	**93.41**	93.30	92.44
AUC	55.40	51.65	86.69	86.31	86.61	**89.84**	85.87
AUC-PR	88.29	83.51	97.23	97.19	97.22	**98.01**	96.89

Bold numbers indicate the best results.

**Table 3:** Statistical significance tests (paired T-test) of the performance difference between the machine learning algorithms and the baselines on stroke case identification.

Baseline	Measure	P Values between the Machine Learning Algorithms and the Baselines				
		LR	SVM-P	SVM-R	RF	ANN
ICD9	ACC	1.04E-12*	1.63E-12*	6.82E-13*	1.65E-12*	9.61E-12*
	P	4.99E-4*	4.95E-6*	7.01E-6*	7.68E-9*	4.67E-7*
	R	3.47E-13*	1.40E-12*	2.50E-13*	9.98E-13*	4.34E-12*
	F	1.50E-12*	2.07E-12*	8.01E-13*	2.31E-12*	9.13E-12*
	AUC	4.40E-11*	3.46E-12*	3.11E-12*	8.43E-12*	1.58E-11*
	AUC-PR	8.28E-12*	1.25E-11*	1.01E-11*	2.46E-12*	1.02E-11*
CLIN	ACC	1.63E-4*	4.65E-5*	1.16E-5*	5.45E-5*	6.96E-4*
	P	1.53E-7*	6.62E-10*	1.43E-10*	1.04E-10*	1.49E-10*
	R	0.999	1.00	0.999	1.00	1.00
	F	1.00E-3*	7.85E-4*	1.13E-4*	3.90E-3*	1.48E-2*
	AUC	4.40E-11*	1.50E-11*	7.37E-12*	1.23E-11*	4.86E-11*
	AUC-PR	5.94E-9*	3.25E-9*	3.80E-9*	3.94E-9*	9.13E-9*

*indicates statistical significance (p value < 0.05).

### Performance of stroke subtype detection

**Fig 6:**
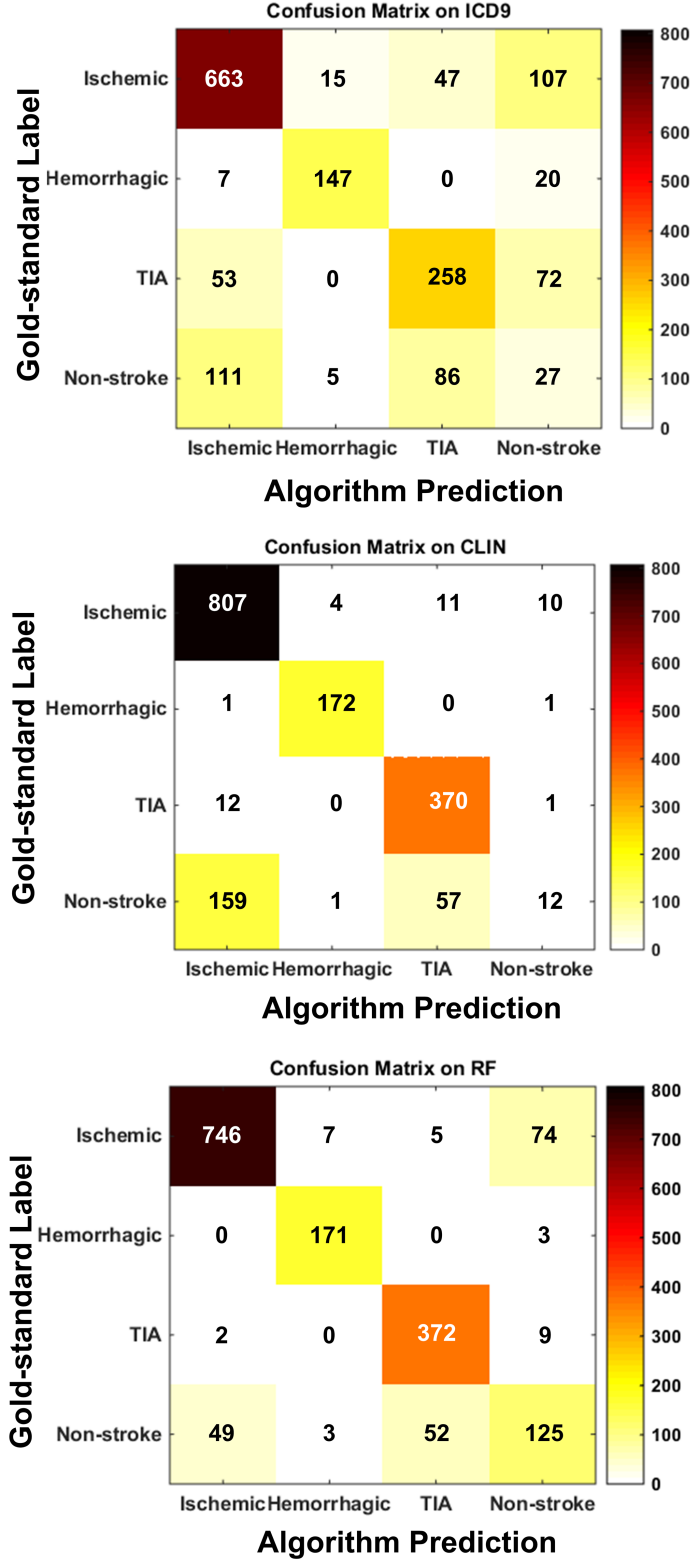
Confusion matrices generated by ICD9, CLIN, and RF on the test set.

Table 4 shows the algorithm performance on stroke subtype detection, where the statistical significance tests were reported in Table 5 on overall accuracy and per-class precision that were of most interest to our study. The improvements of ML classifiers over ICD9 were statistically significant. The ML classifiers also outperformed CLIN significantly on accuracy, and on precisions for ischemic stroke, TIA, and non-stroke “control”. The RF achieved the highest accuracy, and its improvements over the other classifiers were statistically significant (p value <0.05 under paired t-test). Fig 6 illustrates the confusion matrices generated by ICD9, CLIN, and the best-performing RF on the test set, in which an off-diagonal cell (i,j) numbers the events in category i that were misclassified into category j. A more diagonal matrix suggests a more accurate match between algorithm predictions and gold-standard labels.

**Table 4:** Performance of different classification algorithms for stroke type identification.

**Category**	**Measure**	**Cross Validation Performance [%]**						
		**ICD9**	**CLIN**	**LR**	**SVM-P**	**SVM-R**	**RF**	**ANN**
**Overall**	ACC	68.67	84.05	86.66	85.63	86.57	**86.90**	85.75
**Ischemic Stroke**	P	80.64	83.36	89.37	**93.87**	92.22	92.58	90.31
	R	79.59	**97.57**	94.17	88.48	91.05	90.53	91.80
	F	80.11	89.91	**91.70**	91.08	91.62	91.54	91.04
**Hemorrhagic Stroke**	P	87.40	93.88	94.31	**94.68**	93.85	92.69	94.61
	R	82.80	**98.85**	96.97	94.36	96.24	97.98	94.51
	F	84.99	**96.27**	95.60	94.50	95.00	95.23	94.50
**Transient Ischemic Attack**	P	67.42	83.83	87.02	**88.67**	87.43	86.16	88.00
	R	72.47	**96.60**	94.36	89.19	93.18	96.07	91.48
	F	69.81	89.75	90.53	88.90	90.19	**90.84**	89.69
**Non-stroke Control**	P	12.52	31.23	**60.70**	52.30	57.00	59.18	54.67
	R	11.90	2.72	38.71	**62.64**	51.91	49.96	47.55
	F	12.18	4.98	47.10	**56.89**	54.20	54.15	50.75
**Category**	**Measure**	**Test Set Performance [%]**						
		**ICD9**	**CLIN**	**LR**	**SVM-P**	**SVM-R**	**RF**	**ANN**
**Overall**	ACC	67.68	84.12	86.40	85.17	87.08	**87.39**	87.08
**Ischemic Stroke**	P	79.50	82.43	89.91	**94.58**	93.28	93.60	91.16
	R	79.69	**97.00**	92.07	86.06	90.02	89.66	91.71
	F	79.59	89.12	90.97	90.12	**91.62**	91.59	91.43
**Hemorrhagic Stroke**	P	88.02	**97.18**	97.14	95.98	95.53	94.48	94.35
	R	84.48	**98.85**	97.70	95.98	98.28	98.28	95.98
	F	86.22	**98.01**	97.42	95.98	96.88	96.34	95.16
**Transient Ischemic Attack**	P	65.99	84.48	87.65	**89.58**	88.21	86.71	89.22
	R	67.36	96.61	94.52	89.82	93.73	**97.13**	95.04
	F	66.67	90.13	90.96	89.70	90.89	91.63	**92.04**
**Non-stroke Control**	P	11.95	50.00	56.18	50.09	56.77	**59.24**	58.67
	R	11.79	5.24	43.67	**65.54**	56.77	54.59	50.22
	F	11.87	9.49	49.14	56.78	56.77	**56.82**	54.12

Bold numbers indicate the best results.

**Table 5:** Statistical significance tests (paired T-test) of the performance difference between the machine learning algorithms and the baselines on stroke type identification.

Baseline	Measure	P Values between the ML Algorithms and the Baselines				
		LR	SVM-P	SVM-R	RF	ANN
ICD9	Overall ACC	2.23E-10*	2.62E-10*	1.63E-9*	1.65E-10*	3.21E-10*
	P (Ischemic stroke)	6.07E-9*	9.81E-8*	5.24E-8*	1.02E-8*	4.40E-8*
	P (Hemorrhagic stroke)	1.15E-5*	4.73E-5*	2.27E-5*	4.87E-5*	7.92E-5*
	P (TIA)	2.13E-10*	2.17E-9*	7.82E-11*	3.72E-10*	8.84E-11*
	P (Non-stroke control)	2.63E-9*	8.46E-9*	1.61E-8*	8.88E-10*	2.19E-9*
CLIN	Overall ACC	2.40E-3*	1.89E-2*	1.64E-4*	7.57E-7*	2.60E-3*
	P (Ischemic stroke)	4.35E-11*	3.64E-9*	3.94E-9*	1.73E-10*	5.33E-9*
	P (Hemorrhagic stroke)	0.104	0.229	0.529	0.993	0.116
	P (TIA)	3.33E-5*	6.05E-5*	4.56E-6*	2.34E-5*	1.33E-4*
	P (Non-stroke control)	1.90E-3*	2.90E-3*	7.05E-4*	3.14E-4*	2.00E-3*

*indicates statistical significance (p value < 0.05).

## Discussion

Despite being the most common approach for recording clinical conditions, the ICD-9 methods are sub-optimal for phenotyping diseases including stroke.[24] All ML algorithms performed better than ICD9 significantly for stroke case detection. The RF achieved the best performance in terms of the primary measure (Table 2). Its performance was also comparable to that of trained research nurses (CLIN), with better tradeoff between precision and recall (evidenced by the higher AUC-PRs achieved). Both ICD9 and CLIN did not achieve a precision of 95% (Fig 5), and thus their predictions could not be utilized directly to support genetic and genomic research.[72, 73] In comparison, the best-performing RF could provide approximately 90% of the cases with 95% precision (dotted line in Fig 5), which would allow high statistical power for subsequent studies without labor-intensive clinician review.

For stroke subtype detection, the precisions obtained by the algorithms varied between subtypes, with the best on hemorrhagic stroke, followed by ischemic stroke and TIA (Table 4). The variation of performance was in accordance with complexities in diagnosing these stroke subtypes: if a stroke is caused by hemorrhage, a CT scan can show evidence immediately.[40] However, a normal CT scan does not rule out the diagnosis of ischemic stroke and a MRI, particularly diffusion-weighted imaging, is typically required to confirm the findings.[41] Finally, the MRI shows diagnostic findings in a low percentage of TIA cases.[39] Determining TIA additionally relies on a patient’s ability to provide a history of transient stroke-like symptoms, and on a physician’s ability to match these symptoms to the operational concept of TIA. Consequently, the clinical diagnosis of TIA is difficult and has limited inter-observer reliability.[74]

The experimental results (Fig 6 and Table 4) suggested that such complexities in stroke diagnosis affected the baselines and ML algorithms differently. Without comprehensive information from patient records, the ICD9 baseline was unable to distinguish among stroke subtypes accurately. The research nurses were capable of identifying hemorrhagic stroke, but they tended to overcall more complicated subtypes (as evidenced by its confusion matrix). Compared with humans, the confusion matrix made by RF showed fewer misclassifications between ischemic stroke, TIA and non-stroke “control”. In fact, the RF showed comparable performance on detecting hemorrhagic stroke and significantly better precisions on all other categories (Table 4). The findings suggested the strength of ML-based methods in capturing and weighing information from different aspects of patient data to detect stroke subtypes.

In addition, the feature selection process identified a subset of predictive variables that synthesizes a human-oriented conceptualization of stroke diagnosis. The majority of the variables were related to diagnostic tests for stroke (CT/MRI, ANG and CU in Fig 4), and patients’ physiological characteristics during hospitalization (SS and ED).[75] Interventions (IT) such as carotid endarterectomies were used for stroke prevention and they could imply higher risk of stroke onset. Finally, patients’ demographics (DEMO) and substance use behaviors (SU) were shown to be influential, which were consistent with the literature findings.[6, 11, 20] In particular, the CT/MRI and SS were the most predictive variables and they yielded more than 98% of the performance gain (Fig 4). The relative importance of these variables could help physicians weigh the information when chart reviewing a patient’s record.

Our findings contribute to the body of knowledge in stroke research on several fronts. In the experiments the ML models were evaluated on a population from multiple hospitals, and the positive results suggested their generalizability in stroke detection. As such, the developed approach has potential to facilitate case identification for multi-site genomic studies.[72, 73] By leveraging a centralized dataset, a coordinating center could develop and disseminate ML models along with data abstraction protocols. The participating sites could then abstract site-specific data and apply the models to identify stroke cases. The feature selection uncovered a subset of predictive variables (CT/MRI and SS) that could facilitate the development of more effective phenotyping algorithms to reduce workload in data abstraction, which is an interesting direction of our future work. In addition, the ML approach has potential to generalize to other applications in stroke research. Currently, stroke epidemiology studies that utilize administrative databases suffer from misclassification bias by using only ICD discharge codes, whereas the studies involving manual inspection such as the GCNKSS are hindered by time required for data collection and adjudication. By calibrating the ML predictions with linear regression, we could estimate incidence rates of stroke in a study region with a high degree of confidence.[76] As such, the developed approach could provide great benefit for reducing time and effort for executing stroke-related epidemiology studies, allowing near real-time estimates of stroke incidence.

### Error analysis, limitations and future work

We performed error analysis for the RF algorithm on stroke subtype detection. The algorithm made 204 errors on the test data. By reviewing physicians’ clarification on these events, we grouped the errors into nine categories. Table 6 shows the error categories and the numbers of misclassifications between gold-standard and predicted labels for each category. Approximately 42% of errors were due to missing information in the data (categories 1–4). For some events the CT/MRI tests were not performed, possibly due to that the patients did not have stroke symptoms and hence the tests were determined unnecessary by healthcare providers (category 1). The observation suggested that interaction between variables (e.g., no symptoms plus no diagnostic tests) could be informative for stroke diagnosis. In the future we will explicitly model variable connections with tensor product representation and see if it improves the accuracy in stroke detection.[77] In addition, some findings were stored in textual data fields and not used in the current study (category 2). Utilizing NLP algorithms to extract information from these fields is therefore another direction of our future work. Finally, important information was missed occasionally due to healthcare settings (e.g., outpatient) and difficulty of abstraction (e.g., missing subtle information from clinical notes) (categories 3–4). This observation could benefit the design of a more effective abstraction protocol, which however, is out of scope of this study.

**Table 6:** Misclassification errors made by the RF algorithm on the test set.

**ID**	**Gold-standard label**	**IS**			**HS**	**TIA**		**NS**		
	**Predicted label**	**HS**	**TIA**	**NS**	**NS**	**IS**	**NS**	**IS**	**HS**	**TIA**
1	No focal symptoms and key diagnostic tests (CT/MRI findings) were not performed (16.67%)	0	0	2	0	0	0	12	0	20
2	Missing CT/MRI findings (e.g., “no acute intracranial abnormality”) stored in textual data fields (11.27%)	0	0	6	1	0	1	1	0	14
3	Physicians used information not in the data (e.g., raw MRI images and clinical notes) to make the decisions (6.86%)	0	0	7	0	1	1	3	1	1
4	Missing information (e.g., MRI findings) due to ED or outpatient settings (6.86%)	0	1	4	1	0	2	2	0	4
5	Dilemma samples. Physicians determined as cases but the patients did not meet all inclusion criteria. The events were labeled as non-stroke “control” in our study (14.71%)	0	0	0	0	0	0	27	2	1
6	Complex cases. Ischemic stroke with hemorrhagic conversion (4.90%)	7	0	3	0	0	0	0	0	0
7	Undetermined etiology of cases. No focal symptoms or findings from diagnostic tests (12.25%)	0	4	16	1	0	4	0	0	0
8	Conflict findings between symptoms and diagnostic tests (21.08%)	0	0	33	0	1	1	4	0	4
9	Wrong predictions. Unidentified reason (5.39%)	0	0	2	0	0	0	1	0	8

IS: Ischemic stroke; HS: Hemorrhagic stroke; TIA: Transient ischemic attack; NS: non-stroke control. Percentage of errors for each category is presented in the bracket.

Another 32% of errors were ascribed to the complexity of events (categories 5–8). The algorithm identified physicians’ decisions well but did not capture more rigid inclusion criteria, hence misclassifying a noticeable amount of non-stroke “controls” into cases (category 5). It also confused between ischemic and hemorrhagic strokes when the events were ischemic strokes with hemorrhagic conversion (category 6). To solve these problems, we plan to implement knowledge-based post-processing to explicitly include structured inclusions and to adjust algorithm predictions when patients present both ischemic and hemorrhagic characteristics. In addition, the algorithm misclassified several ischemic events with unknown etiology, in which the patients did not present traditional stroke symptoms and diagnostic findings (category 7). Understanding the etiology of these events will help identify predictors for the ML-based methods, which warrants further investigation by neurologists.

Finally, approximately 21% of errors were caused by conflicts between patients’ symptoms and diagnostic findings (mainly CT/MRI findings). If a patient had focal stroke symptoms but CT/MRI findings were normal, the study physicians often override the findings and considered the patient a case because the symptoms could be mild such that they did not show up on CT/MRI. In contrast, if there were multiple CT/MRI tests showing consistent findings, the physicians would weigh more on diagnostic results even if the patient did not have symptoms. Compared with stroke physicians, the RF algorithm always weighted more on CT/MRI findings and had less flexibility in balancing conflict variables, consequently misclassifying a notable amount of events in which the CT/MRI findings were normal. To alleviate this problem, we will develop advanced multi-layer classifiers in our future work to balance weights between different variable sets before aggregating them for stroke detection.[47]

One significant limitation of the study is that the variables used were abstracted by research nurses. Manual abstraction of clinical variables requires not only substantial subspecialty expertise, but also intensive manpower. Consequently, the limitation could hinder the dissemination of the developed approach across institutions. To alleviate this problem, variables should ideally be extracted from EHR data automatically. Recent studies have shown the feasibility of automating abstraction of stroke related risk factors from EHR data.[78, 79] Because the ML models could achieve competitive performance with a limited set of 44 variables (CT/MRI and SS), we anticipate that automated data abstraction for stroke detection is feasible with appropriate NLP and regular expression algorithms.

Another limitation is that we did not assess the inter-observer reliabilities among stroke physicians in the epidemiology study. Although each hospitalization event was reviewed by at least one clinical nurse and a stroke physician, and the complex events were adjudicated through group discussion, variability may exist in the final adjudications, particularly for TIA and stroke cases with negative diffusion-weighted imaging results. To address this limitation, we have initiated documentation of physician decisions in the ongoing GCNKSS, which allows for the evaluation of inter-observer reliabilities on future data. In addition, we grouped the stroke subtypes into four categories to avoid the problem of data sparseness. To improve the granularity of detection, we will continue collecting data from the GCNKSS to develop more powerful predictive models.

As a final limitation, the work was limited to reporting system performance on a population collected in a single epidemiology study. To assess its generalizability, project planning is in progress to evaluate the developed approach in a separate stroke population with different data collection and representation methods.

## Conclusions

In this study we demonstrated the strength of ML technologies in identifying stroke cases and pathological subtypes. By analyzing a broad array of patient data, the ML models showed good capacity for detecting stroke diagnosis. The algorithms significantly outperformed the ICD-9 method that is commonly implemented in current studies. Their performance was comparable to that of trained research nurses, with better tradeoff between precision and recall. The feature selection uncovered a subset of predictive variables, which could facilitate future development of effective stroke phenotyping algorithms. The anticipated benefits of machine learning have potential to bring stroke phenotyping to the forefront of biomedical research, unlocking high statistical power for subsequent genetic and genomic studies.

## Supporting information

S1 Table Description of the variables used in the study. (DOCX)
