# Supplementary material for: Towards phenotyping stroke: Leveraging data from a large-scale epidemiological study to detect stroke diagnosis
Source: PLoS One. 2018 Feb 14;13(2):e0192586. doi: 10.1371/journal.pone.0192586 (PMC5812624; doi:10.1371/journal.pone.0192586)
Supplement: S1 Table — (DOCX) [file pone.0192586.s001.docx]

# Appendix

**S1 Table.** Description of the variables used in the study.

| **Variable Category** | **Variable Description** | **Variable Value** |
| --- | --- | --- |
| DEMO | Age | Numerical value |
| DEMO | Sex | 1. Male 2. Female |
| DEMO | Race | 1. White 2. Black 3. Asian 4. Native Hawaiian/Pacific Islander 5. American Indian/Alaskan Native 6. Other 7. Unknown |
| DEMO | Ethnicity | 1. Hispanic 2. Non-Hispanic 3. Undocumented |
| DEMO | Marital status | 1. Single 2. Married 3. Living with partner 4. Widowed 5. Divorced 6. Separated 7. Unknown |
| DEMO | Employment status | 1. Employed 2. Unemployed 3. Retired 4. Disabled 5. Homemaker 6. Unknown |
| SU | Smoking use | 1. Yes 2. No 3. Unknown |
| SU | Years of smoking | Numerical value |
| SU | Number of packs per day | Numerical value |
| SU | Current smoker (smoked within last 3 months) | 1. Yes 2. No |
| SU | Alcohol use | 1. Yes 2. No 3. Unknown |
| SU | Patient noted on EHR as heavy drinker, defined as more than 2 servings per day | 1. Yes 2. No 3. Binge drinking 4. Former alcoholic 5. Unknown |
| SU | Use of marijuana | 1. Yes 2. No 3. Unknown |
| SU | Use of cocaine/crack | 1. Yes 2. No 3. Unknown |
| SU | Alcohol detected in urine or blood | 1. Yes 2. No 3. Unknown |
| SU | Drug detected in urine or blood | 1. Yes 2. No 3. Unknown |
| SU | Street drug use within 24 hours of admission | 1. Yes 2. No 3. Unknown |
| VI | Type of healthcare institution | 1. Acute hospital 2. Autopsy 3. Outpatient clinic 4. Physician 5. Nursing home 6. Emergency Department (ED) only 7. Other |
| VI | Residence at time of admission | 1. Home 2. Nursing home 3. Assisted living 4. Other 5. Unknown |
| VI | Site where symptoms occurred | 1. Residence 2. Work 3. Hospital 4. Other 5. Unknown |
| VI | First medical contact | 1. 911 2. Primary medical doctor (visit) 3. Primary medical doctor (phone) 4. NH/assisted living 5. Hospital 6. ED 7. Coroner 8. Unknown |
| ED | ED encounter | 1. Yes 2. No, in-house event 3. Yes, but no record 4. Direct admit 5. Onset occurred in ED 6. Other 7. Unknown |
| ED | Sent to CT/MRI | 1. Yes 2. No 3. Unknown |
| ED | Disposition from ED | 1. Admit 2. Discharged home 3. Transfer to another hospital 4. Expired 5. Left AMA 6. Other |
| ED | Diagnosis from ED chart | 1. stroke/CVA/TIA 2. MI 3. Seizure 4. Fall/found down/unresponsive 5. Weakness/numbness 6. Headache/migraine 7. Other |
| ED | Glasgow coma score (eyes) | 1-4 |
| ED | Glasgow coma score (verbal) | 1-5 |
| ED | Glasgow coma score (motor) | 1-6 |
| ED | Glasgow coma score (total) | 3-15 |
| ED | Endovascular treatment | 1. Yes 2. No |
| ED | Arterial line | 1. Yes 2. No |
| ED | Use of antihypertensive medication | 1. Yes 2. No |
| ED | ED Blood pressure (Systolic) | Numerical value |
| ED | ED Blood pressure (Diastolic) | Numerical value |
| SE | Is time of symptoms known? | 1. Yes 2. No |
| SE | If no, estimated time of symptoms onset | 1. Awoke with symptoms 2. >24 hours onset time 3. After midnight 4. Morning 5. Afternoon 6. Evening 7. Unknown |
| SE | Blood pressure if no ED record (Systolic) | Numerical value |
| SE | Blood pressure if no ED record (Diastolic) | Numerical value |
| SE | Modified Rankin Scale prior to stroke/TIA onset | 1. No symptoms 2. No significant disability despite SXs; able to carry out usual activities 3. Slight disabilities; unable to carry out all previous activities, but able to look after self without assistance 4. Moderate disabilities; requiring some help; able to walk without assistance 5. Moderately severe disabilities; unable to walk without assistance; unable to attend to body needs 6. Severe disability; bedridden; incontinent; requiring constant nursing care 7. Not available |
| SE | Does patient use a cane (documented)? | 1. Yes 2. No 3. Unknown |
| SE | Does patient use a walker (documented)? | 1. Yes 2. No 3. Unknown |
| SE | Evaluated by a neurologist? | 1. Yes 2. No 3. Unknown |
| SE | Evaluated by stroke team? | 1. Yes 2. No 3. Unknown |
| SE | Evaluated by a neurosurgeon? | 1. Yes 2. No 3. Unknown |
| SE | NIH stroke scale done by stroke team? | 1. Yes 2. No 3. Unknown |
| SE | NIH stroke scale (total score) | Numerical value |
| SE | Record type of NIH stroke scale | 1. Prospective 2. Retrospective 3. Not done |
| SE | Evaluation documentation utilized from | 1. Stroke team 2. ED documentation 3. Neurologist 4. Primary care provider 5. Other |
| SE | Level of consciousness (LOC) | 1. Alert, keenly responsive 2. Not alert, arousable by minor stimulation to obey, answer, respond 3. Not alert, requires repeated or painful stimulation to attend 4. Responds only with reflex motor or autonomic effects, or totally unresponsive, flaccid, areflexic |
| SE | LOC questions | 1. Oriented x 3 2. Oriented x <3 3. disoriented; if receptive deficit present and orientation not reported, code as disoriented |
| SE | LOC commands | 1. Notation of “follows commands” and no receptive deficit noted 2. Any intermediate description with or without receptive deficit 3. Notation of “does not follow commands,” with or without receptive deficit |
| SE | Best gaze | 1. Normal 2. Partial gaze palsy; gaze abnormal in one or both eyes, but forced deviation of total gaze paresis not present 3. Forced deviation or total gaze paresis not overcome by oculocephalic maneuver |
| SE | Visual | 1. No visual loss 2. Partial hemianopia 3. Complete hemianopia |
| SE | Facial palsy | 1. Normal symmetrical movement 2. Minor paralysis (flattened NLF, asymmetry on smiling) 3. Partial paralysis (total or near-total paralysis of lower face) 4. Complete paralysis (absence of movement in upper and lower face) |
| SE | Left arm | 1. MRC 4+ or 5 2. MRC 4 or “mild” 3. MRC 3 4. MRC 2 5. MRC 0 or 1 |
| SE | Right arm | 1. MRC 4+ or 5 2. MRC 4 or “mild” 3. MRC 3 4. MRC 2 5. MRC 0 or 1 |
| SE | Left leg | 1. MRC 4+ or 5 2. MRC 4 or “mild” 3. MRC 3 4. MRC 2 5. MRC 0 or 1 |
| SE | Right leg | 1. MRC 4+ or 5 2. MRC 4 or “mild” 3. MRC 3 4. MRC 2 5. MRC 0 or 1 |
| SE | Limb ataxia | 1. Absent 2. Present in one limb 2. Present in two limbs |
| SE | Sensory | 1. Normal; no sensory loss 2. Mild to moderate sensory loss 3. Severe to total sensory loss |
| SE | Best language | 1. No aphasia, normal 2. Expressive deficit only 3. Expressive plus receptive deficit or receptive deficit only 4. Mute, global aphasia; no usable speech or auditory comprehension |
| SE | Dysarthria | 1. Normal 2. Mild to moderate; patient slurs at least some words and, at worst, can be understood with some difficulty 3. Severe; patient’s speech is so slurred as to be unintelligible in the absence of or out of proportion to any dysphasia, or is mute/anarthric |
| SE | Neglect | 1. No abnormality 2. Visual, tactile, auditory, spatial, or personal inattention or extinction to bilateral simultaneous stimulation in one of the sensory modalities 3. Profound hemi-inattention or hemi-inattention to >1 modality; does not recognize own hand or orients to only one side of space |
| SS | Weakness | 1. Yes 2. No 3. Generalized 4. Unknown |
| SS | Weakness (face) | 1. Left 2. Right 3. Both 4. Not seen 5. Unspecified 6. Unknown |
| SS | Weakness (arm) | 1. Left 2. Right 3. Both 4. Not seen 5. Unspecified 6. Unknown |
| SS | Weakness (leg) | 1. Left 2. Right 3. Both 4. Not seen 5. Unspecified 6. Unknown |
| SS | Numbness (sensory loss) | 1. Yes 2. No 3. Generalized 4. Unknown |
| SS | Numbness (face) | 1. Left 2. Right 3. Both 4. Not seen 5. Unspecified 6. Unknown |
| SS | Numbness (arm) | 1. Left 2. Right 3. Both 4. Not seen 5. Unspecified 6. Unknown |
| SS | Numbness (leg) | 1. Left 2. Right 3. Both 4. Not seen 5. Unspecified 6. Unknown |
| SS | Headache | 1. Yes 2. No 3. Unknown |
| SS | Mental status | 1. Alert and orient 2. Drowsy/somnolent 3. Coma/unresponsive 4. Posturing 5. Alert but confused 6. Alerted mental status (unspecified) 7. Unknown |
| SS | Speech | 1. Normal 2. Slurred/dysarthric 3. Aphasic 4. Mute 5. Dysarthric & aphasic 6. Abnormal, unspecified 7. Other 8. Unknown |
| SS | Fall/cannot walk | 1. Yes 2. No 3. Unknown |
| SS | Vision | 1. Normal 2. Blurred 3. Double 4. Partial loss 5. Total blindness 6. Photophobia 7. Other 8. Unknown |
| SS | Dizzy/vertigo | 1. Yes 2. No 3. Unknown |
| SS | Ataxia | 1. Yes 2. No 3. Unknown |
| SS | Dysphagia/drooling | 1. Yes 2. No 3. Unknown |
| SS | Nausea/vomiting | 1. Yes 2. No 3. Unknown |
| SS | Seizure/jerking | 1. Yes 2. No 3. Unknown |
| SS | Other | 1. Yes 2. No |
| SS | Did symptoms last more than 24 hours? | 1. Yes 2. No 3. Other 4. Unknown |
| CT/MRI | CT of head done after onset? | 1. Yes 2. No 3. Unknown |
| CT/MRI | Reason for CT | 1. Initial imaging 2. Routine follow up 3. Clinical decline 4. Other 5. Unknown |
| CT/MRI | Primary finding of CT | 1. Normal 2. Acute infarct 3. ICH 4. SAH 5. AVM 6. IVH 7. Old infarct 8. Subacute infarct 9. Prior hemorrhage 10. Hemorrhage conversion of acute infarct 11. Other 12. Unknown |
| CT/MRI | Secondary finding of CT | 1. Normal 2. Acute infarct 3. ICH 4. SAH 5. AVM 6. IVH 7. Old infarct 8. Subacute infarct 9. Prior hemorrhage 10. Hemorrhage conversion of acute infarct 11. Other 12. Unknown |
| CT/MRI | Third finding of CT | 1. Normal 2. Acute infarct 3. ICH 4. SAH 5. AVM 6. IVH 7. Old infarct 8. Subacute infarct 9. Prior hemorrhage 10. Hemorrhage conversion of acute infarct 11. Other 12. Unknown |
| CT/MRI | Second CT done? | 1. Yes 2. No 3. Unknown |
| CT/MRI | Reason for second CT | 1. Initial imaging 2. Routine follow up 3. Clinical decline 4. Other 5. Unknown |
| CT/MRI | Primary finding of second CT | 1. Normal 2. Acute infarct 3. ICH 4. SAH 5. AVM 6. IVH 7. Old infarct 8. Subacute infarct 9. Prior hemorrhage 10. Hemorrhage conversion of acute infarct 11. Other 12. Unknown |
| CT/MRI | Secondary finding of second CT | 1. Normal 2. Acute infarct 3. ICH 4. SAH 5. AVM 6. IVH 7. Old infarct 8. Subacute infarct 9. Prior hemorrhage 10. Hemorrhage conversion of acute infarct 11. Other 12. Unknown |
| CT/MRI | Third finding of second CT | 1. Normal 2. Acute infarct 3. ICH 4. SAH 5. AVM 6. IVH 7. Old infarct 8. Subacute infarct 9. Prior hemorrhage 10. Hemorrhage conversion of acute infarct 11. Other 12. Unknown |
| CT/MRI | MRI done | 1. Yes 2. No 3. Unknown |
| CT/MRI | Reason for MRI | 1. Initial imaging 2. Routine follow up 3. Clinical decline 4. Other 5. Unknown |
| CT/MRI | Diffusion-weighted imaging? | 1. Yes 2. No 3. Unknown |
| CT/MRI | DWI positive for acute cerebral infarct? | 1. Yes 2. No 3. Unknown |
| CT/MRI | Primary finding of MRI | 1. Normal 2. Acute infarct 3. ICH 4. SAH 5. AVM 6. IVH 7. Old infarct 8. Subacute infarct 9. Prior hemorrhage 10. Hemorrhage conversion of acute infarct 11. Other 12. Unknown |
| CT/MRI | Secondary finding of MRI | 1. Normal 2. Acute infarct 3. ICH 4. SAH 5. AVM 6. IVH 7. Old infarct 8. Subacute infarct 9. Prior hemorrhage 10. Hemorrhage conversion of acute infarct 11. Other 12. Unknown |
| CT/MRI | Third finding of MRI | 1. Normal 2. Acute infarct 3. ICH 4. SAH 5. AVM 6. IVH 7. Old infarct 8. Subacute infarct 9. Prior hemorrhage 10. Hemorrhage conversion of acute infarct 11. Other 12. Unknown |
| CT/MRI | Second MRI done | 1. Yes 2. No 3. Unknown |
| CT/MRI | Reason for second MRI | 1. Initial imaging 2. Routine follow up 3. Clinical decline 4. Other 5. Unknown |
| CT/MRI | Diffusion-weighted imaging? | 1. Yes 2. No 3. Unknown |
| CT/MRI | DWI positive for acute cerebral infarct? | 1. Yes 2. No 3. Unknown |
| CT/MRI | Primary finding of second MRI | 1. Normal 2. Acute infarct 3. ICH 4. SAH 5. AVM 6. IVH 7. Old infarct 8. Subacute infarct 9. Prior hemorrhage 10. Hemorrhage conversion of acute infarct 11. Other 12. Unknown |
| CT/MRI | Secondary finding of second MRI | 1. Normal 2. Acute infarct 3. ICH 4. SAH 5. AVM 6. IVH 7. Old infarct 8. Subacute infarct 9. Prior hemorrhage 10. Hemorrhage conversion of acute infarct 11. Other 12. Unknown |
| CT/MRI | Third finding of second MRI | 1. Normal 2. Acute infarct 3. ICH 4. SAH 5. AVM 6. IVH 7. Old infarct 8. Subacute infarct 9. Prior hemorrhage 10. Hemorrhage conversion of acute infarct 11. Other 12. Unknown |
| ANG | MR angiography done? | 1. Yes 2. No 3. Yes, but no record 4. Unknown |
| ANG | Findings of MR angiography | 1. Normal 2. Abnormal 3. Unknown |
| ANG | CT angiography done? | 1. Yes 2. No 3. Yes, but no record 4. Unknown |
| ANG | Findings of CT angiography | 1. Normal 2. Abnormal 3. Unknown |
| ANG | Cerebral angiography done? | 1. Yes 2. No 3. Yes, but no record 4. Unknown |
| ANG | Findings of cerebral angiography | 1. Normal 2. Abnormal 3. Unknown |
| CU | Carotid ultrasound done | 1. Yes 2. No 3. Yes, but no record 4. Unknown |
| CU | Significant abnormal carotid findings | 1. Yes 2. No 3. Unknown |
| ECHO | Echocardiogram done | 1. Yes 2. No 3. Yes, but no record 4. Unknown |
| ECHO | Type of echocardiogram | 1. Transthoracic 2. Transesophageal 3. Both 4. unknown |
| ECHO | Quality of study | 1. Adequate 2. Poor 3. Limited 4. Unknown |
| ECHO | Abnormal echocardiographic findings | 1. Yes 2. No 3. Unknown |
| ECHO | Left atrial enlargement | 1. Yes 2. No 3. Unknown |
| ECHO | Left ventricular hypertrophy | 1. Yes 2. No 3. Unknown |
| ECHO | Valvular vegetation | 1. Yes 2. No 3. Unknown |
| ECHO | Cardiomyopathy | 1. Yes 2. No 3. Unknown |
| ECHO | Mural thrombus | 1. Yes 2. No 3. Unknown |
| ECHO | Mitral valve prolapse | 1. Yes 2. No 3. Unknown |
| ECHO | Akinetic wall | 1. Yes 2. No 3. Unknown |
| ECHO | Ventricular aneurysm | 1. Yes 2. No 3. Unknown |
| ECHO | LV ejection fraction | 1. Yes 2. No 3. Normal 4. Unknown |
| ECHO | Spontaneous echocardiographic contrast of “smoke” | 1. Yes 2. No 3. Unknown |
| ECHO | Mitral valve stenosis/sclerosis | 1. Yes 2. No 3. Unknown |
| ECHO | Aortic valve stenosis/sclerosis | 1. Yes 2. No 3. Unknown |
| ECHO | Patent foramen ovale | 1. Yes 2. No 3. Unknown |
| ECHO | Atrial septal aneurysm | 1. Yes 2. No 3. Unknown |
| ECHO | Aortic atherosclerotic debris | 1. Yes 2. No 3. Unknown |
| EKG | Admission electrocardiogram done | 1. Yes 2. No 3. Yes, but no record 4. Unknown |
| EKG | Findings of admission EKG | 1. Normal 2. Abnormal 3. Borderline 4. Unknown |
| EKG | Sinus bradycardia < 60 | 1. Yes 2. No |
| EKG | Sinus tachycardia > 100 | 1. Yes 2. No |
| EKG | PAC (Atrial ectopic)/PVC (Ventricular ectopic) | 1. Yes 2. No |
| EKG | Atrial fibrillation/flutter | 1. Yes 2. No |
| EKG | Third degree heart block | 1. Yes 2. No |
| EKG | Left ventricular hypertrophy | 1. Yes 2. No |
| EKG | Myocardial infarctions of indeterminate age | 1. Yes 2. No |
| EKG | Acute myocardial infarctions | 1. Yes 2. No |
| EKG | V-Fib/V-Tach | 1. Yes 2. No |
| EKG | Pauses (>5 seconds) | 1. Yes 2. No |
| EKG | S-T changes/abnormalities | 1. Yes 2. No |
| EKG | Patient diagnosed with atrial fibrillation this hospitalization? | 1. Yes 2. No 3. Unknown |
| EKG | Findings of chest X-ray | 1. Normal 2. Abnormal 3. Not done 4. Unknown |
| EKG | Chest X-ray abnormality | 1. CHF 2. Pneumonia 3. Pleural effusion 4. Other 5. Unknown |
| LAB | White blood cell count | Numerical value |
| LAB | Hemoglobin | Numerical value |
| LAB | Platelet count (in thousands) | Numerical value |
| LAB | Creatinine | Numerical value |
| LAB | Presenting glucose level | Numerical value |
| LAB | Low-density lipoprotein | Numerical value |
| LAB | High-density lipoprotein | Numerical value |
| LAB | Triglycerides | Numerical value |
| LAB | Total cholesterol | Numerical value |
| LAB | Prothrombin time | Numerical value |
| LAB | International normalized ratio | Numerical value |
| LAB | Partial thromboplastin time | Numerical value |
| LAB | Creatine Kinase-MB | Numerical value |
| LAB | Troponin | Numerical value |
| MH | Current Coumadin use | 1. Yes 2. No 3. Unknown |
| MH | Reason of Coumadin use | 1. Atrial fibrillation 2. TIA/stroke 3. Mechanical heart valve 4. Heart failure 5. Deep vein thrombosis 6. Pulmonary embolism 7. Other 8. Unknown |
| MH | History of hypertension | 1. Yes 2. No 3. Unknown |
| MH | Drug treatment for hypertension | 1. Yes 2. No 3. Unknown |
| MH | Diabetes mellitus | 1. Yes 2. No 3. Unknown |
| MH | Under treatment of diabetes mellitus | 1. Yes 2. No 3. Unknown |
| MH | Type of treatment | 1. Diet 2. Oral medications 3. Insulin 4. Unknown |
| MH | History of elevated cholesterol | 1. Yes 2. No 3. Unknown |
| MH | Treatment of elevated cholesterol | 1. Diet 2. Drug therapy 3. None 4. Diet & drug therapy 5. Unknown |
| MH | History of coronary artery disease | 1. Yes 2. No 3. Unknown |
| MH | History of myocardial infarction | 1. Yes 2. No 3. Unknown |
| MH | History of atrial fibrillation by EKG | 1. Yes 2. No 3. Unknown |
| MH | History of angina | 1. Yes 2. No 3. Unknown |
| MH | History of congestive heart failure | 1. Yes 2. No 3. Unknown |
| MH | Baseline ejection fraction | 1. Yes (specified) 2. No 3. Documented as normal 4. Unknown |
| MH | Heart valve replacement | 1. Yes 2. No 3. Unknown |
| MH | Type of heart valve replacement | 1. Biological 2. Mechanical 3. Unknown |
| MH | Site of heart valve replacement | 1. Mitral 2. Aortic 3. Both 4. Pulmonic 5. Tricuspid 6. Unknown |
| MH | Prior cardiac bypass surgery | 1. Yes 2. No 3. Unknown |
| MH | Cardiac vessel angioplasty/stent | 1. Yes 2. No 3. Unknown |
| MH | Cardiac pacemaker | 1. Yes 2. No 3. Unknown |
| MH | Reason for pacemaker | 1. Sick sinus syndrome 2. Third degree heart block 3. Other 4. Unknown |
| MH | Aicd/Defibrillator placed? | 1. Yes 2. No 3. Unknown |
| MH | Cardiomyopathy | 1. Yes 2. No 3. Unknown |
| MH | History of carotid artery disease | 1. Yes 2. No 3. Unknown |
| MH | Carotid endarterectomy | 1. Yes 2. No 3. Unknown |
| MH | Side of most recent endarterectomy | 1. Right 2. Left 3. Both 4. Unknown |
| MH | Carotid stenting/angioplasty | 1. Yes 2. No 3. Unknown |
| MH | Most recent side involved in carotid stenting/angioplasty | 1. Right 2. Left 3. Unknown |
| MH | Surgery/procedure within the last 30 days? | 1. Yes 2. No 3. Unknown |
| MH | Cerebral angiogram within 24 hours prior to onset? | 1. Yes 2. No 3. Unknown |
| MH | Coronary angiogram within 24 hours prior to onset? | 1. Yes 2. No 3. Unknown |
| MH | Thrombolytic therapy received for reason other than stroke/TIA within 24 hours prior to onset | 1. Yes 2. No 3. Unknown |
| MH | Recent emboli to peripheral arteries (in last 3 months, to leg, arm, kidney, GI system) | 1. Yes 2. No 3. Unknown |
| MH | Recent deep vein thrombosis | 1. Yes 2. No 3. Unknown |
| MH | Greenfield filter | 1. Yes 2. No 3. Unknown |
| MH | History of peripheral vascular disease | 1. Yes 2. No 3. Unknown |
| MH | Dementia | 1. Yes 2. No 3. Unknown |
| MH | Type of dementia | 1. Alzheimer’s 2. Multi-infarct 3. Other 4. Unknown |
| MH | Depression | 1. Yes 2. No 3. Unknown |
| MH | Sickle cell disease | 1. Yes 2. No 3. Unknown |
| MH | Hemophilia | 1. Yes 2. No 3. Unknown |
| MH | HIV positive | 1. Yes 2. No 3. Unknown |
| MH | History of brain tumor | 1. Yes 2. No 3. Unknown |
| MH | History of malignancy | 1. Yes 2. No 3. Unknown |
| MH | History of seizure | 1. Yes 2. No 3. Unknown |
| MH | History of migraine | 1. Yes 2. No 3. Unknown |
| MH | Infection within the last 2 weeks | 1. Yes 2. No 3. Unknown |
| MH | If so, type of infection | 1. Upper respiratory infection 2. Pneumonia 3. Urinary tract infection 4. Other 5. Unknown |
| MH | Other significant medical conditions | 1. Yes 2. No 3. Unknown |
| MH | Influenza vaccine within the last year | 1. Yes 2. No 3. Unknown |
| MH | Pneumonia vaccine (within the last 5 years) | 1. Yes 2. No 3. Unknown |
| SH | Prior history of stroke | 1. Yes 2. No 3. Unknown |
| SH | Number of prior strokes | Numerical value |
| SH | Any residual impairment from prior strokes | 1. Yes 2. No 3. Unknown |
| SH | Type of most recent stroke | 1. Cerebral Infarct 2. ICH 3. SAH 4. IVH 5. Unknown |
| SH | Cerebral aneurysm | 1. Yes 2. No 3. Unknown |
| SH | Arteriovenous malformation | 1. Yes 2. No 3. Unknown |
| SH | TIA | 1. Yes 2. No 3. Unknown |
| SH | Any first degree relative ever had ischemic stroke | 1. Yes 2. No 3. Unknown |
| SH | Any first degree relative ever had TIA | 1. Yes 2. No 3. Unknown |
| SH | Any first degree relative ever had ICH | 1. Yes 2. No 3. Unknown |
| SH | Any first degree relative ever had SAH | 1. Yes 2. No 3. Unknown |
| SH | Any first degree relative ever had stroke (unknown type) | 1. Yes 2. No 3. Unknown |
| SH | Any first degree relative ever had Alzheimer’s disease or dementia | 1. Yes 2. No 3. Unknown |
| SH | Any first degree relative ever had brain aneurysm | 1. Yes 2. No 3. Unknown |
| SH | Any first degree relative ever had hypertension | 1. Yes 2. No 3. Unknown |
| SH | Any first degree relative ever had diabetes | 1. Yes 2. No 3. Unknown |
| SH | Any first degree relative ever had hypercholesterolemia | 1. Yes 2. No 3. Unknown |
| SH | Any first degree relative ever had coronary artery disease | 1. Yes 2. No 3. Unknown |
| ICD9 | Primary and secondary ICD-9 codes on patients’ discharge lists | 1. 430.x-438.x 2. Not recorded |
| DX | Anemia | 1. Yes 2. No |
| DX | Anxiety | 1. Yes 2. No |
| DX | Arrhythmias | 1. Yes 2. No |
| DX | Bleeding | 1. Yes 2. No |
| DX | Bowel/bladder dysfunction | 1. Yes 2. No |
| DX | Brain edema | 1. Yes 2. No |
| DX | Cardiac arrest | 1. Yes 2. No |
| DX | Cellulitis | 1. Yes 2. No |
| DX | Chest pain / angina | 1. Yes 2. No |
| DX | CHF / pulmonary edema | 1. Yes 2. No |
| DX | Confusion / agitation | 1. Yes 2. No |
| DX | Decubitus ulcer | 1. Yes 2. No |
| DX | Dehydration | 1. Yes 2. No |
| DX | Depression (newly diagnosed) | 1. Yes 2. No |
| DX | Dizziness | 1. Yes 2. No |
| DX | Deep vein thrombosis | 1. Yes 2. No |
| DX | Dysphagia (not requiring peg/ng) | 1. Yes 2. No |
| DX | Dysphagia (requiring ng) | 1. Yes 2. No |
| DX | Dysphagia, (requiring peg) | 1. Yes 2. No |
| DX | Fall/injury | 1. Yes 2. No |
| DX | Fever | 1. Yes 2. No |
| DX | GI bleed | 1. Yes 2. No |
| DX | Hallucinations | 1. Yes 2. No |
| DX | Headaches | 1. Yes 2. No |
| DX | Herniation (documented) | 1. Yes 2. No |
| DX | Hyperglycemia | 1. Yes 2. No |
| DX | Hypertensive crisis | 1. Yes 2. No |
| DX | Hypoglycemia | 1. Yes 2. No |
| DX | Hypotensive episode | 1. Yes 2. No |
| DX | Infection | 1. Yes 2. No |
| DX | Myocardial infarction | 1. Yes 2. No |
| DX | Nausea and vomiting | 1. Yes 2. No |
| DX | Pain | 1. Yes 2. No |
| DX | PEG removal | 1. Yes 2. No |
| DX | Pneumonia | 1. Yes 2. No |
| DX | Pulmonary edema | 1. Yes 2. No |
| DX | Pulmonary embolus | 1. Yes 2. No |
| DX | Rash | 1. Yes 2. No |
| DX | Renal failure | 1. Yes 2. No |
| DX | Respiratory failure | 1. Yes 2. No |
| DX | Seizure | 1. Yes 2. No |
| DX | Sepsis | 1. Yes 2. No |
| DX | Trach insertion | 1. Yes 2. No |
| DX | Trach removal | 1. Yes 2. No |
| DX | Upper respiratory infection | 1. Yes 2. No |
| DX | Urinary tract infection | 1. Yes 2. No |
| DX | Other diagnosis | 1. Yes 2. No |
| IT | Major surgeries (following and related to neurological event) | 1. Yes 2. No 3. Unknown |
| IT | Type of surgery | 1. Aneurysm clipping 2. Clot evaluation 3. AVM resection 4. Hemicraniectomy 5. Carotid endarterectomy 6. Carotid stent placement 7. Aneurysm coiling 8. Other |
| IT | Intubation | 1. Yes 2. No |
| IT | Intraventricular drain or shunt placed | 1. Yes 2. No |
| IT | IV GTT medication for hypertension control | 1. Yes 2. No |
| IT | IV GTT medications for hypotension control | 1. Yes 2. No |
| IT | Blood pressure closest to symptom onset (Systolic) | Numerical value |
| IT | Blood pressure closest to symptom onset (Diastolic) | Numerical value |
| IT | Was TPA administered following onset of stroke? | 1. Yes (for stroke) 2. No 3. Yes, other than stroke |
| IT | Was other thrombolytic therapy given following onset of stroke? | 1. Yes (for stroke) 2. No 3. Yes, other than stroke |
| IT | IV heparin following ischemic event | 1. Yes 2. No 3. Unknown |
| IT | Nimodipine following SAH | 1. Yes 2. No 3. Unknown |
| IT | Other experimental drug | 1. Yes 2. No 3. Unknown |
| TH | DVT prophylaxis | 1. Yes 2. No 3. Not documented 4. Ambulatory 5. Unknown 6. N/A |
| TH | LMW heparinoid | 1. Yes 2. No 3. Unknown 4. N/A |
| TH | Sequential compression device | 1. Yes 2. No 3. Unknown 4. N/A |
| TH | Ted hose | 1. Yes 2. No 3. Unknown 4. N/A |
| TH | Other prevention used | 1. Yes 2. No 3. Unknown 4. N/A |
| TH | Foley catheter | 1. Yes 2. No 3. Unknown 4. N/A |
| TH | Sliding scale insulin (ordered) | 1. Yes 2. No 3. Unknown 4. N/A |
| TH | Swallowing evaluation | 1. Yes 2. No 3. Unknown 4. N/A |
| TH | Smoking cessation intervention | 1. Yes 2. No 3. Unknown 4. N/A |
| TH | Physical therapy evaluation | 1. Yes 2. No 3. Unknown 4. N/A |
| TH | Occupational therapy evaluation | 1. Yes 2. No 3. Unknown 4. N/A |
| TH | Speech therapy evaluation | 1. Yes 2. No 3. Unknown 4. N/A |
| TH | Physical therapy | 1. Yes 2. No 3. Unknown 4. N/A |
| TH | Occupational therapy | 1. Yes 2. No 3. Unknown 4. N/A |
| TH | Speech therapy | 1. Yes 2. No 3. Unknown 4. N/A |
| O | Vital status at final discharge | 1. Dead 2. Alive |
| O | Cause of death | 1. Complications of CVA 2. Cardiac death 3. No initiation of care 4. Withdrawal of care 5. Other comorbidity 6. Other 7. Unknown |
| O | Modified Rankin Scale | 1. No symptoms 2. No significant disability despite SXs; able to carry out usual activities 3. Slight disabilities; unable to carry out all previous activities, but able to look after self without assistance 4. Moderate disabilities; requiring some help; able to walk without assistance 5. Moderately severe disabilities; unable to walk without assistance; unable to attend to body needs 6. Severe disability; bedridden; incontinent; requiring constant nursing care 7. Not available |
| O | Does patient use a cane (documented)? | 1. Yes 2. No 3. Unknown 4. N/A |
| O | Does patient use a walker (documented)? | 1. Yes 2. No 3. Unknown 4. N/A |
| O | Disposition at final discharge | 1. Home 2. Relative or friend 3. Rehabilitation 4. Skilled nursing facility 5. Assisted living 6. Hospital/Acute setting 7. Other 8. Unknown |
